# When We May, and When We May Not, Bleed

**Published:** 1876-08

**Authors:** 


When we May, and when we May Not, Bleed.—Dr. Henry Moon thinks that the indiscriminate use of blood-letting, years ago, destroyed many thousand lives annually. In some cases, however, with the light of modern science, it is still a remedy of great practical usefulness. In cerebral hemorrhage, if the impulse of the heart be strong and its sounds loud, if the pulse be regular, and no signs of œdema of the lungs exist, the patient should be bled without delay. Here a judicious, timely bleeding may prevent the extension of the paralysis from the cerebrum to the medulla oblongata. The patient may not be bled, on the contrary, when the heart's action is weak, the pulse irregular, and rattling in the trachea has already begun. In acute croupous pneumonia, bleeding may be beneficial when the pneumonia has attacked a vigorous and hitherto healthy person, the temperature being higher than one hundred deg. F., and the pulse rating at more than one hundred and twenty beats in a minute. In those, however, who are already debilitated, bleeding increases the danger of exhaustion. Should the fever in pneumonia be moderate, venesection is not indicated, even in healthy and vigorous individuals. The result of a bold venesection in fluxion of the lung, arising from excessive cardiac action, is astonishing. As soon as the volume of the blood has become lessened, the pressure diminishes in the arteries, the patients often breathing more freely during the operation. The bloody foam which they are expectorating vanishes, and life may be rescued from the greatest danger by aid of the physician. Bleeding should never be used in cases of long standing disease of the heart, except under the most pressing circumstances. In endocarditis, bleeding is decidedly injurious, yet at times a condition exists, such as overcharge of the pulmonary circulation, threatening œdema of the lungs, when prompt relief by diminution of the blood is demanded.—*British Medical Journal.*

